# Protein kinase C and human uterine contractility

**DOI:** 10.1186/1471-2393-7-S1-S11

**Published:** 2007-06-01

**Authors:** Isabelle Eude-Le Parco, Emmanuelle Dallot, Michelle Breuiller-Fouché

**Affiliations:** 1 Institut Jacques Monod, CNRS UMR7592, Université Paris 6 et 7, Paris, F-75006, France; 2 INSERM U767, Paris, F-75006, France; 3 Université René Descartes, Paris, F-75006, France

## Abstract

Abnormalities in uterine contractility are thought to contribute to several clinical problems, including preterm labor. A better understanding of the mechanisms controlling uterine activity would make it possible to propose more appropriate and effective management practices than those currently in use. Recent advances point to a role of the protein kinase C (PRKC) family in the regulation of uterine smooth muscle contraction at the end of pregnancy. In this review, we highlight recent work that explores the involvement of individual PRKC isoforms in cellular process, with an emphasis on the properties of PRKCZ isoform.

## Background

**Figure 1:**
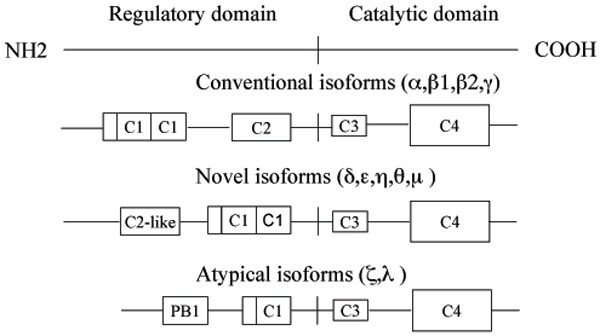
**Diagrammatic representation of the domain structure of PRKC isoforms**. Conventional, cPRKC isoforms (PRKCA, PRKCB1, PRKCB2, and PRKCG also known as PRKCα,β1,β2, and γ, respectively) are diacylglycerol (DAG) sensitive and Ca2+ responsive (C2 is the Ca2+-binding domain, C1 features the DAG and phosphatidylserine binding; at the N-terminal of the C1 domain is located a pseudosubstrate site that regulates PRKC activity). Novel, nPRKC isoforms (PRKCD, PRKCE, PRKCH, PRKCQ, and PRCKM also known as PRKC δ,ε,η,θ, and μ, respectively) are DAG sensitive but Ca2+ insensitive (the C2-like domain do not retain Ca2+-coordinating residues). Atypical, aPRKC isoforms (PRKCZ and PRKCI, also known as PRKC ζ and λ, respectively) are regulated by phospholipidic mediators products including phosphatidylinositol 3,4,5-triphosphate). All kinases have a conserved kinase core (C4) and a C3 domain, which represents the ATP-binding site (for a review see [12].

During human pregnancy, development and contractile activity of uterine smooth muscle are regulated by numerous maternal and fetal signals. Steroid hormones, growth factors, catecholamines, eicosanoids, neuropeptides, and cytokines have all been implicated in the growth and differentiation of the myometrium as well as in its contractility. The past 17 yr have seen the emergence of a new potent vasoconstrictor, endothelin-1 (EDN1, abbreviated as ET-1) that can exert a wide spectrum of biological actions in many different tissues including uterine smooth muscle (for a review see [1]). The concentration of ET-1 in maternal blood is increased during pregnancy and reaches a peak at term, and its concentration in amniotic fluid is also high [2]. Like other uterotonic agents, ET-1 is effective for stimulating the force and frequency of human myometrial contractions in vitro. The uterine contractile responsiveness to ET-1 is markedly increased at the end of pregnancy [3] and is accompanied by an elevation of the density of myometrial endothelin receptor type A (EDNRA) receptors functionally coupled to the phospholipase C (PLC)/calcium system [4,5]. Activation of the polyphosphoinositide signaling cascade plays a key role during generation of ET-1-stimulated myometrial contractions. Among the multigene family of serine/threonine kinases, the protein kinase C (PRKC) has been shown to participate to the regulation of uterine smooth muscle contraction, but species-related differences in PRKC-mediated signaling have been reported [6-10]. A total of 11 isoforms have been cloned to date, and they are classified in three groups on the basis of their cofactor requirements [11,12] (Figure 1).

## Results and discussion

### A role for PRKC in ET-1-induced uterine contraction at the end of pregnancy

At least three conventional PRKC isoforms (cPRKC) (PRKCA, PRKCB1, PRKCB2), two novel PRKC isoforms (nPRKC) (PRKCD, PRKCE) and an atypical PRKCZ isoform were detected in human pregnant myometrium [13,14].

**Figure 2:**
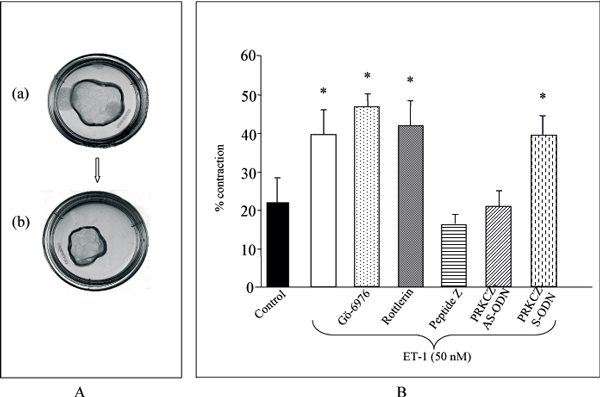
**A-ET-1 induces contraction of collagen lattices by myometrial cells**. Photographs shown are of free-floating collagen lattices in culture dishes (diameter 35 mm). Collagen lattices are shown at time t = 0 (a) and after incubating for 24 h in serum-free containing 50 nM ET-1 (b). B-Effect of PRKC inhibition on ET-1-promoted gel contraction. Free-floating collagen lattices were incubated without (control) or with the cPRKC inhibitor Gö-6976 (0.5 μM), the PRKCD inhibitor Rottlerin (3 μM), the inhibitory peptide specific for PRKCZ peptide Z, the antisense oligonucleotide (AS-ODN) (4 μM) or sense (S-ODN) directed against PRKCZ before the addition of 50 nM ET-1. Results are expressed as mean percentage contraction ± SE of at least 3 determinations. *P < 0.05 compared with control.

It seems to be less obvious whether PRKC plays a role in the kinase cascade involved in the myometrial contractile process during human pregnancy. We have attempted to individualize the PRKC isoform able to selectively elicit uterine contraction at the end of pregnancy. Selective inhibitors of cPRKC and PRKCD isoforms, do not prevent ET-1-induced contractions, whereas LY294002, a phosphoinositide-3-kinase (PI3K) inhibitor, affects the contractile response [13,15]. These results suggest but not demonstrate a role for aPRKC isoforms in myometrial contractions. We extended the analysis to a complementary model of cultured myometrial cells, which could be utilized for identifying potential regulatory pathways that modulate normal uterine activity. In a functional assay using collagen gel retraction, we confirmed that human myometrial cells maintain a smooth muscle cell phenotype in culture and responded appropriately to the potent uterotonic agent, ET-1, via selective activation of the EDNRA receptor [16]. This technique was therefore utilized to elucidate the precise contribution of the PRKC pathways in myometrial cell contraction. As shown in Figure 2, pharmacological inhibition of cPRKC and nPRKC did not affect myometrial cell contraction. Treatment of myometrial cell lattices with an inhibitory peptide specific for PRKCZ or with an antisense-oligonucleotide (AS-ODN) against PRKCZ resulted in a significant lost of ET-1-induced contraction. In addition, activated PRKCZ associates with the actin component of the cytoskeleton of cultured human pregnant myometrial cells and may regulate the phosphorylation or binding properties of actin. These different approaches provide the first evidence that only PRKCZ activation mediated ET-1-induced myometrial contraction in late pregnancy [15]. In contrast, in one study, Ozaki *et al. *[17] reported that PRKC activation by phorbol ester possibly through PRKCB pathway, enhances contraction induced by high K+ in the pregnant human myometrium with increasing Ca2+ sensitivity of contractile elements, while a report by Bermeo *et al*. [18] found that magnesium sulfate and oxytocin require extracellular calcium to induce activation of both PRKCA and PRKCD in cultured myometrial cells from pregnant women. The controversial role of PRKC isoforms in uterine contractility required clarification.

### Role of aPRKC and NFKB in human myometrial cells

The control of the onset of labor in women is as yet unknown, but the implication of the PRKCZ in the contractile uterine process at the time of parturition is not so surprising.

Why? Modulation of the immune/inflammatory mechanisms, specially a balance between T helper (Th1)/Th2 cytokine production, has been described at the time of parturition, and a link between TNF/IL1B and premature human childbirth has been proposed [19]. Additionally, aPRKCs are important components of the TNF/IL1B signaling pathway that control NFKB activation [20-22]. Undoubtedly, it is important to elucidate the probable interaction between these aPRKC isoforms and the cascade of pro-inflammatory cytokines.

Approximately 20% to 33% of preterm birth is associated with an underlying infection process. Systemic or local intrauterine infections have been implicated in the pathogenesis of preterm labor and delivery (for a review, see [23]). Exposure to microbial insult induces preterm birth in several animal models. For example in the pregnant mouse, the endotoxin lipopolysaccharide (LPS) (a glycolipid derived from the outer membrane of gram-negative bacteria)-enhances amplitude of *in vitro *spontaneous uterine contractile activity [24] and premature labor and delivery can be induced *in vivo *by both local and systemic administration of LPS [25-27].

In humans, bacterial vaginosis and intrauterine infection with Gram-negative are now believed to be an important factor of risk for preterm labor [28]. The invasion of microorganisms in the utero-genital sphere triggers a release of pro-inflammatory cytokines such as TNF/IL1B. Activation of the cytokine cascade results in the production of uterotonic agents leading to parturition. Resident macrophages are thought to be the principal cell type in the female genital tract with a receptor-mediated signal transduction mechanism that is essential for immune responses to LPS [29].

**Figure 3:**
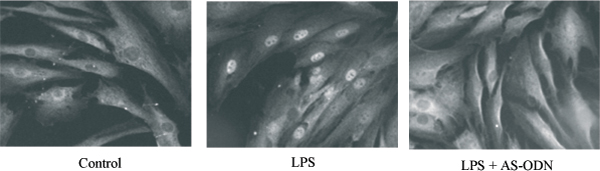
**Inhibition of PRKCZ prevents the nuclear translocation of NFKB p65 subunit induced by LPS in myometrial cells**. Cells were treated without (control) or for 120 min with LPS (10 μg/ml) in the absence or presence of AS-ODN (4 μM) directed against human PRKCZmRNA. Cells were fixed, permeabilized and immunostained with antibody against NFKB p65 subunit followed by anti-rabbit FITC (magnification × 400).

Further analysis has been conducted on a model of bacterial LPS-induced inflammation of myometrial cells in order to examine a potential role of PRKCZ in this process. This *in vitro *model using LPS should mimic human infection-associated preterm labor. Myometrial cells express TLR4 receptors and have the potential to respond to LPS. This is further supported by the activation of PRKCZ by LPS and by the fact that LPS is able to activate the NFKB pathway. As shown in Figure 3, incubation of human myometrial cells with AS-ODN to PRKCZ abolished the LPS-induced nuclear translocation of NFKB p65 subunit [30]. It is therefore conceivable that NFKB p65 signalling pathway includes PRKCZ in our cell model. Unraveling the whole cascade of NFKB activation occurring in myometrial cells remains a difficult challenge. The enigma becomes complex, because of a switch in NFKB subunit composition in the human myometrium during pregnancy and labor [31]. In addition, increased expression of NFKB in the human myometrium at end of pregnancy is involved in functional progesterone withdrawal [32]. A question arising from these observations is whether activated NFKB also increases expression of target genes that cause increased myometrial contractility. One plausible candidate is the cyclooxygenase-2 (also known as prostaglandin-endoperoxide synthase 2, PTGS2) that might be included among the members of the "cassette of contraction-associated proteins". Recently, Duggan *et al*. [33] have established that aPRKCs mediate NFKB transcriptional activation and form part of the signaling network required for IL1B-induced PTGS2 expression in human cultured myometrial cells.

## Conclusion

All these results indicate that the aPRKC-related pathway is involved in the uterine contractile process at the end of pregnancy. Future work is needed to elucidate the interactions between aPRKC isoform and the cascade of cytokines, but the results reported here unveil a novel mechanism that links aPRKC to inflammatory signaling in the myometrium. Knowledge of the precise mechanism of aPRKCs (PRKCZ?) in the cascade of events supporting the preterm labor in human, will open the development of novel strategies to prevent premature delivery.

## List of abbreviations

PRKC, protein kinase C; PRKCA, protein kinase Cα; PRKCB, protein kinase Cβ; PRKCD, protein kinase Cδ; PRKCE, protein kinase Cε; PRKCZ, protein kinase Cζ; EDNRA, endothelin receptor type A; PTGS2, prostaglandin-endoperoxide synthase 2; LPS, bacterial endotoxin lipopolysaccharide; PI3K, phosphoinositide-3-kinase.

## Competing interests

The authors declare that they have no competing interests.

## Authors' contributions

MBF conceived of the study, and participated in its design and coordination and helped to draft the final report. ED contributed to all the steps of the study and prepared the illustrations. IEL contributed to the design of the study and discussion of the results. All authors read and approved the final manuscript.
